# The eMERGE Network: A consortium of biorepositories linked to electronic medical records data for conducting genomic studies

**DOI:** 10.1186/1755-8794-4-13

**Published:** 2011-01-26

**Authors:** Catherine A McCarty, Rex L Chisholm, Christopher G Chute, Iftikhar J Kullo, Gail P Jarvik, Eric B Larson, Rongling Li, Daniel R Masys, Marylyn D Ritchie, Dan M Roden, Jeffery P Struewing, Wendy A Wolf

**Affiliations:** 1 Center for Human Genetics, Marshfield Clinic Research Foundation, 1000 North Oak Avenue (MLR), Marshfield, WI, 54449, USA; 2 Center for Genetic Medicine, Northwestern University, 303 East Superior Street, Chicago, IL, 60611, USA; 3 Division of Biomedical Statistics and Informatics, Mayo Clinic, 200 First Street SW, Rochester, MN 55905, USA; 4 Division of Cardiovascular Diseases, Mayo Clinic, 200 First Street SW, Rochester, MN, 55905, USA; 5 Department of Genome Sciences, University of Washington, 3720 15th Ave NE, Seattle WA 98195, USA; 6 Group Health Research Institute, 1730 Minor Avenue, Suite 1600, Seattle, WA, 98101, USA; 7 Office of Population Genomics, National Human Genome Research Institute, 5635 Fishers Lane, Suite 3058, MSC 9307, Bethesda, MD, 20892-9307, USA; 8 Department of Biomedical Informatics, Vanderbilt University School of Medicine, Room 416 Eskind Medical Library, Nashville, TN, 37232, USA; 9 Center for Human Genetics Research, Vanderbilt University School of Medicine; 519 Light Hall, Nashville, IN 37231, USA; 10 Department of Pharmacology, Vanderbilt University School of Medicine, 1285 Medical Research Building IV, Nashville, TN, 37232, USA

## Abstract

**Introduction:**

The eMERGE (electronic MEdical Records and GEnomics) Network is an NHGRI-supported consortium of five institutions to explore the utility of DNA repositories coupled to Electronic Medical Record (EMR) systems for advancing discovery in genome science. eMERGE also includes a special emphasis on the ethical, legal and social issues related to these endeavors.

**Organization:**

The five sites are supported by an Administrative Coordinating Center. Setting of network goals is initiated by working groups: (1) Genomics, (2) Informatics, and (3) Consent & Community Consultation, which also includes active participation by investigators outside the eMERGE funded sites, and (4) Return of Results Oversight Committee. The Steering Committee, comprised of site PIs and representatives and NHGRI staff, meet three times per year, once per year with the External Scientific Panel.

**Current progress:**

The primary site-specific phenotypes for which samples have undergone genome-wide association study (GWAS) genotyping are cataract and HDL, dementia, electrocardiographic QRS duration, peripheral arterial disease, and type 2 diabetes. A GWAS is also being undertaken for resistant hypertension in ≈2,000 additional samples identified across the network sites, to be added to data available for samples already genotyped. Funded by ARRA supplements, secondary phenotypes have been added at all sites to leverage the genotyping data, and hypothyroidism is being analyzed as a cross-network phenotype. Results are being posted in dbGaP. Other key eMERGE activities include evaluation of the issues associated with cross-site deployment of common algorithms to identify cases and controls in EMRs, data privacy of genomic and clinically-derived data, developing approaches for large-scale meta-analysis of GWAS data across five sites, and a community consultation and consent initiative at each site.

**Future activities:**

Plans are underway to expand the network in diversity of populations and incorporation of GWAS findings into clinical care.

**Summary:**

By combining advanced clinical informatics, genome science, and community consultation, eMERGE represents a first step in the development of data-driven approaches to incorporate genomic information into routine healthcare delivery.

## Background

The mapping of the human genome has enabled new exploration of how genetic variations contribute to health and disease. To better realize this promise, the National Human Genome Research Institute (NHGRI) called on researchers to determine how genetic variants influence susceptibility towards chronic conditions such as diabetes, Alzheimer's disease, and cardiovascular disease, in order to ultimately improve patient care [1]. This effort has been remarkably successful [2]. The results of this work form the cornerstone not only for discovery of new biologic pathways and drug targets, but also for enabling a vision of "personalized medicine" in which genomic (and other personal health information) is incorporated into the fabric of healthcare. Delivering such advances to the bedside will require advanced information technology, based on electronic medical record (EMR) systems. Each of the five sites in the eMERGE network, formed to address these challenges, includes a DNA repository linked to an EMR, and thus the network is acting as a test bed for enabling such a future vision of healthcare. Further, aggregation of results across the network could represent an initial step toward the generation of a large prospective cohort in which healthcare status linked to biosamples are used to evaluate the impact of both genes and environment [3] on important human phenotypes, i.e. a trans-institutional or ultimately a national biobank. There are many challenges, however, to the successful execution of this idea: examples include validation and harmonization of phenotypes identified using medical records data, integration of genomic and phenotypic data across multiple sites for combined GWAS analysis, and adequacy of informed consent to share GWAS data widely, such as in the database of genotypes and phenotypes (dbGaP) [4], managed by the NIH.

The stated goals of the eMERGE Network (http://www.gwas.net) are to develop and apply approaches for using U.S. biorepositories linked to EMR systems for large-scale genomic research by determining: 1) the completeness and validity of phenotypic and exposure information derived from EMR; 2) the adequacy of existing consent for sharing data widely with other investigators within the network and outside the network through dbGaP; 3) the needs for additional consent and/or consultation with biorepository participants, investigators and other relevant groups; 4) best practices for IRB interactions, participant consent, and results reporting, and for collecting, documenting and sharing data; 5) representativeness and diversity of biorepository participants; and 6) associations of genome-wide data with EMR-defined phenotypes. Each center participating in the consortium, organized by the NHGRI with additional funding from the National Institute of General Medical Sciences, proposed to study the relationship between genome-wide genetic variation and a common disease/trait. In addition, the consortium includes a focus on ethical issues such as privacy, confidentiality, and interactions with the broader community.

## Network Structure, Aims and Progress

All sites proposed a specific disease-related GWAS with specific aims related to validation of electronic phenotyping and community consultation. At each site, identification of ~3,000 phenotype appropriate subjects was to be followed by a GWAS in that set, as well as a 6^th ^GWAS (~2,000 samples) accrued across the network. To identify subjects for study, electronic algorithms are developed, validated, and modified if necessary until positive predictive values of at least 95% are achieved where possible. All sites committed to making their electronic algorithms and NLP tools widely available and to sharing GWAS data for validated cases and controls for the other network site outcomes.

### Network Organization

**Figure 1:**
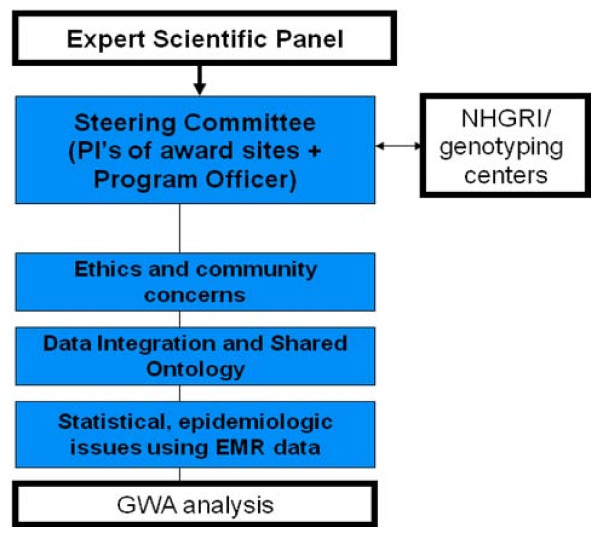
Organizational structure of the eMERGE network

The network was formed in fall 2007, and its organizational structure is shown in Figure 1. The steering committee (Principal and other investigators and NHGRI staff) meets 2-3 times annually. Setting of network goals is initiated by the working groups: (1) Genomics, (2) Informatics, (3) Consent & Community Consultation Working Group, which also includes active participation by investigators outside the eMERGE funded sites, and (4) Return of Results Oversight Committee. A five-member External Scientific Panel (ESP) was formed to provide independent input to the NHGRI director about the progress and direction of the Network and meets annually with the Steering Committee.

#### Administrative Coordinating Center

The eMERGE Network Administrative Coordinating Center (ACC), at Vanderbilt University Medical Center, provides a range of support to the network including coordination of steering committee meetings, organization of and support for working groups, a communications hub with public and private pages (http://www.gwas.net), and support for the ESP. In addition to administrative support, the ACC has assumed responsibilities for coordination of informatics and genomics efforts across sites. These include: 1) developing methods to compare the clinical characteristics of populations with specific disease phenotypes identified across institutions in the network; 2) providing standardized quality control for genotypes generated across the network and 3) Data Privacy Consultation service to assist in quantifying the risk of re-identification of de-identified data, e.g., by comparison of sets of diagnostic codes to publicly available Medicare data, health and vital statistics registries, and voter lists. The ACC received a supplement to provide standardized QA/QC for the genotypic data for all sites.

### Site-specific Biorepository Descriptions and Aims

There are five sites in eMERGE: Marshfield, Northwestern, Mayo, Group Health Seattle, and Vanderbilt. In the following sections, the biobank at each institution is first described, followed by a brief description of the phenotypes being studied and community consultation activities. Key features of each biobank are summarized in Table 1.

**Table 1:** Comparison of Biobanks and Phenotypes Across the eMERGE sites

**Site**	**Biobank design**	**Biobank size and demographics**	**Phenotypic outcomes**
Marshfield	Population-based	20,000; 98% Caucasian, mean age 48, range 18-102	Low HDL cholesterol, cataract (n = 3968) Secondary: diabetic retinopathy
Mayo Clinic	PAD cases identified from the Mayo non-invasive vascular laboratory database; control subjects without PAD identified from the Cardiovascular Health Clinic	1687 cases (mean age 65) and 1725 controls (mean age 60)	Peripheral arterial disease (PAD) (n = 3412); Secondary: red blood cell indices
Northwestern	Outpatient clinic and hospital-based	Approximately 10,000; 12% African American, 8% Hispanic; Mean age 50, range 18 - 90+	Primary: type II diabetes (n = 3531); Secondary: lipids and height
Group Health	ACT Study Cohort of aged 65 and olderrandomly sampled from an HMO all known not to be demented at enrollment and followed for development of dementia, and Alzheimer's Disease (source of cases and controls) ADPR: Alzheimer's disease cases from a model incidence case registry (source of cases)	Approximately 4000 persons over age 65 from ACT Study	Alzheimer Disease (n = 3390), carotid artery stenosis; Secondary: statin adverse events
Vanderbilt	Use of discarded blood/non-human subjects linked to electronic medical records	Approximately 75,000; 70% Caucasian, 10% African American; mean age 53, range 18-100	Electrocardiographic QRS duration (n = 3192); Secondary: PheWAS

#### Marshfield Clinic Research Foundation

##### Biobank description

The Marshfield Clinic Personalized Medicine Research Project (PMRP) is a population-based biobank with approximately 20,000 adult participants and access to an average of 30 years of medical history data [5-7]. DNA, plasma, and serum samples are stored, with written informed consent from participants to allow sharing of de-identified samples and data. At the time of enrollment, subjects complete a brief questionnaire with demographic and personal behavior data including smoking and alcohol intake, and detailed food frequency questionnaire.

##### eMERGE project aims

*Phenotypes*

Marshfield is developing and validating two primary outcomes: high-density lipoprotein (HDL) and cataract and quantifying the impact of two environmental factors (cigarette smoking and statin use) known to influence each of the primary study outcomes. Age-related cataract is the leading cause of blindness globally and low HDL cholesterol levels are a risk factor for myocardial infarction, the leading cause of mortality in the US. No GWAS data are available for cataract and no gene/environment GWAS data are available for HDL cholesterol levels. In addition to an epidemiologic association and shared risk factors, cataract and low HDL cholesterols levels were selected because of the suspected difficulty to develop validated electronic algorithms. Cataract was expected to be the most difficult because Ophthalmology was the last Department at the Marshfield Clinic to use the EMR and because much of the components of an ophthalmic exam necessary to define phenotype are not gathered in structured format and would require more sophisticated electronic tools, including natural language processing (NLP). In addition to NLP, intelligent character recognition (ICR) is being used to identify cataract type from the clinical notes.

Another outcome, diabetic retinopathy, was selected for funding through an ARRA supplement to mine the phenotypic data available for subjects being genotyped for the primary outcomes. Diabetic retinopathy was selected for several reasons. First, this outcome leverages the primary outcome of diabetes in the eMERGE network, as well as leveraging cases and controls from other sites to improve statistical power. Second, diabetic retinopathy is the leading cause of vision impairment in working aged adults and there have been no GWAS data published on this outcome.

*Community consultation*

Quarterly meetings of the PMRP Community Advisory Group and quarterly newsletters are used to engage and inform the public about this GWAS and the sharing of data with the wider research community. The external Ethics and Security Advisory Board, chaired by Norman Fost from the University of Wisconsin, was reconvened in the context of eMERGE to advise on the updates to the consent form. After revising the existing consent form document to be more explicit about data sharing into dbGaP and making other changes to reflect protocol changes since the original consent form was written and approved in 2002, a computer-based consenting process is being developed and evaluated. Built-in questions to evaluate knowledge and understanding will allow investigators to confirm informed consent prior to enrollment.

#### Northwestern University

##### Biobank description

The Northwestern University biorepository, NUgene (http://www.nugene.org), is a clinic- and hospital-based biobank with approximately 10,000 adult participants from the patient population at the Northwestern University Medical Center (NUMC) [8,9]. Participants' DNA samples are coupled with data from an enrollment questionnaire and longitudinal data from the EMR representing actual clinical care events. NUgene has access to participants' clinical care data via a consolidated data warehouse. Participants consent to distribution and use of their coded DNA samples and data for a broad range of genetic research conducted by third-party investigators. Supplementing the EMR data, participants complete a standardized enrollment questionnaire that captures self-reported demographics, race and ethnicity, selected environmental exposure data, individual health history, and family history of disease. The NUgene population is representative of five-county Chicago area (Cook, DuPage, Kane, Lake, McHenry, and Will counties) based on comparisons of sex, age, race and ethnicity with census data. NUgene represents a clinically diverse population, including samples and data from healthy individuals and patients with common adult onset conditions.

NUMC has been operating comprehensive, commercial EMR systems for both inpatient and outpatient populations for over 10 years. Phenotypic data on NUgene participants is mined through Northwestern's Enterprise Data Warehouse (EDW) which consolidates clinical data from all patients receiving care at NUMC. Established to enable clinical and translational research, the EDW currently stores over 2 million patient records.

##### eMERGE project aims

*Phenotypes*

Northwestern's primary phenotype for eMERGE focuses on type 2 diabetes. Multiple GWAS for type 2 diabetes have been completed and susceptibility variants have been identified [10], providing known targets to test the hypothesis that EMR-derived phenotypes can be used as an alternative approach to identify variants associated with disease. Northwestern is developing and validating computational algorithms to identify cases and controls from NUgene, and other eMERGE populations, for type 2 diabetes GWAS. Approaches are also being developed to extract variables for known type 2 diabetes confounders. The eMERGE type 2 diabetes GWAS population is comprised of samples from NUgene and BioVU at Vanderbilt University, and focuses case and control selection to maximally include participants of African American ancestry.

Through the Northwestern ARRA supplement, investigators are leveraging available GWAS and EMR data across the network to study serum lipid levels (total cholesterol, HDL, LDL, and triglycerides) as continuous traits, as well as height. To date, published GWAS have only explained a small amount of the variance among lipid traits which are known to be influenced to different degrees by environmental factors. These factors will be addressed across the network.

*Community Consultation*

Since its inception, the NUgene Project has involved advisors from the NUMC scientific, medical, and ethics community as well as external advisors consisting of community members, community engaged researchers and public health experts. Through eMERGE, the focus of these consultations is on issues surrounding consent for GWAS and data sharing with dbGaP and other network investigators. The Northwestern University IRB has played a pivotal role in providing guidance surrounding consent for GWAS and appropriate means to inform participants about data sharing. NUgene continues to engage the community advisory committee on GWAS and data sharing as well as providing additional information to participants through a newsletter. Northwestern's eMERGE community engagement research consists of a 3-phased approach to learning about participant and public concerns about data sharing and obtaining appropriate consent for GWAS [11], engaging IRBs about data sharing issues, and holding consensus meetings with professional stakeholders to determine appropriateness of our consent process for GWAS and data sharing.

#### Mayo Clinic

##### Biobank description

The Mayo biobank is a disease-specific biobank for peripheral arterial disease (PAD). PAD patients were identified from individuals referred to the non-invasive vascular laboratory for lower extremity arterial evaluation. Since 1997, laboratory findings have been recorded into an electronic database employing an in-house software package for data archiving and retrieval; this data becomes part of the Mayo EMR. Patients referred to the center with suspected PAD undergo a comprehensive non-invasive evaluation including the ankle-brachial index (ABI) - the ratio of blood pressure measured in the upper arms divided by blood pressure measured at the ankles. Controls subjects are identified from patients referred to the Cardiovascular Health Clinic for screening for cardiovascular disease. A large proportion of these patients has undergone exercise ECG to rule out coronary artery disease. Data regarding risk factors for atherosclerosis such as diabetes, dyslipidemia, hypertension, and smoking are ascertained from the EMR.

##### eMERGE project aims

*Phenotypes*

For the eMERGE project, the Mayo site chose PAD, a relatively prevalent disease affecting ~8 million adults in the US including nearly 20% of the elderly (>70 years of age). PAD is associated with significant mortality and morbidity [12,13]. However not much is known about the genetic bases of PAD even though several GWAS for coronary artery disease have been completed and multiple susceptibility variants identified. The Mayo GWAS for PAD will complement current understanding of the spectrum of genetic variants associated with atherosclerotic vascular disease. Additional analyses are attempting to understand how environmental and lifestyle measures (e.g., smoking), identified from the Mayo EMR, modify the observed relationship between genotype and the atherosclerotic vascular disease phenotypes (i.e., gene-environment interaction).

The specific aim of the Mayo supplementary proposal is to identify genomic loci influencing red blood cell (RBC) indices using a GWAS approach in cohorts of the eMERGE network. The RBC indices include RBC count, hemoglobin level, mean corpuscular volume, mean corpuscular hemoglobin, RBC distribution width and erythrocyte sedimentation rate. The findings from the proposed analyses will help to characterize molecular mechanisms underlying inter-individual variability in RBC indices, provide novel insights into anemia and related hematologic diseases, and could eventually contribute to the development of new therapeutic approaches for such diseases.

*Community Consultation*

Since genomic data cannot easily or with certainty be fully de-identified or anonymized, an important aim of the Mayo eMERGE project is to engage extensively with research participants and the community regarding best practices to weigh the future benefits of genomic research to patients, families, and the society against the potential risks. The investigators will develop and refine consenting procedures in collaboration with Mayo's IRB on the basis of these findings, through an "Ethics Incubator" developed as part of Mayo's Clinical and Translational Science Award (CTSA). A combination of in-depth patient interviews, consenting "experiments", and community engagement using Deliberative Democracy methods are being employed.

#### Group Health Cooperative, University of Washington and the Fred Hutchinson Cancer Research Center

##### Biobank description

The Group Health (GH) biobank for eMERGE is based within an established and growing cohort of 3,793 patients recruited within GH and actively followed for Alzheimer's Disease (AD) and dementia. Two sub-samples from GH form the specific population analyzed in this project. The Alzheimer's Disease Patient Registry (ADPR) [14] was established in 1986. A related study, Genetic Differences, allowed collection of DNA for persons in the ADPR. The ADPR was complemented in 1994-1996 and ultimately succeeded by the prospective Adult Changes in Thought (ACT) study [15]. Both ADPR and ACT were initiated - and continue in their 23^rd ^year - with funding from a U01 from the National Institute on Aging. ADPR enrolled 695 dementia cases from GH. ACT enrolled 2,581 dementia-free individuals in 1994-1996, with 811 additional dementia-free individuals from 2000-2002, and has since 2005 been enrolling dementia-free subjects continuously with identical methods to keep at least 2000 person-years under observation at all times. DNA is available on 95% of the cohort. The ACT sub-sample is well-characterized, with research assessments every 2 years of risk factors, cognition, blood pressure, physical performance, over-the-counter medication use, and other research-quality study data on both phenotypes and environmental exposures. The ACT sub-sample is stable; for the original cohort, median enrollment in GH was 19 years prior to joining the ACT study, and 85% of the cohort has ≥10 years of GH enrollment. This is a useful cohort for population-based genome-wide GWA studies and offers the somewhat unique characteristic of very long-term longitudinal data. The cohort also has noteworthy value as controls for subsequent GWA analyses of other phenotypes, as it is well-characterized, and many participants have lived to advanced old age under continuous observation.

##### eMERGE project aims

*Phenotypes*

Group Health's primary phenotype of interest is Alzheimer's Disease (AD) using dementia cases and controls within the GH biorepository, based on gold-standard research diagnoses. A 2-stage screening process was used to identify cases in ACT. The Cognitive Abilities Screening Instrument (CASI) [16] is a cognitive test derived from the Mini-Mental State Examination (MMSE) [17]. The CASI is administered to all ACT participants every 2 years. CASI scores ≤85 prompt a dementia evaluation; for comparison purposes, in the Honolulu Asia Aging Study (HAAS) the CASI cut point is 74. Informant, subject, or staff reports of cognitive difficulties also trigger evaluation. The 2^nd ^stage diagnostic examination has two parts: neuropsychological testing and a neurological exam by a physician. Medical records are abstracted for standardized labs (complete blood counts, chemistry panel, B_12_, thyroid stimulating hormone) and neuroimaging. If any of these are unavailable in the prior year they are obtained. These data are used to complete standard DSM-IV diagnostic criteria for dementia and subtypes, NINCDS-ADRDA criteria for AD, and 3 sets of criteria for vascular dementia. The dementia neuropsychological battery includes tests of clock drawing, verbal fluency, Mattis Dementia Rating Scale, Boston naming, verbal paired associations and recall, logical memory and recall, Word List Memory, Constructional Praxis and recall, Trails A and B, and Information and Comprehension subtest items. All clinical data are reviewed at a consensus conference. These procedures have been used continuously over 23 years since the inception of the ADPR in 1986. ACT dementia and AD diagnoses have been used in many papers, including an incidence rate paper, and high-impact papers on exercise^18^, cholesterol^19^, statins^20^, and vitamins^21^.

Group Health used its "gold standard" dementia cases and controls to create and validate an EMR-based dementia definition. Development of the definition included examination of a number of ICD-9 codes for Alzheimer's disease, number of codes plus an "event" (neuroimaging, B12, TSH assays), codes by specialty providers vs. generalists, and fills for dementia drugs. The best criterion found for all-cause dementia was ≥ 5 ICD-9 codes for Alzheimer's Disease and/or ≥ 1 fill for a dementia medication. This EMR definition has a sensitivity of 55% and positive predictive values of 73%. The EMR definition is being used to identify additional dementia cases from other participating sites. White blood cell (WBC) indices are being studied as secondary phenotypes with ARRA supplement funds.

*Community Consultation*

The University of Washington and Group Health have also implemented a consensus process with key stakeholders to develop recommendations concerning consent, data sharing, and return of research results to subjects. Stakeholders will include ACT subjects, GH patients, GH leadership, and ACT investigators. The consensus process is being informed by targeted focus group data collection from ACT subjects, GH enrollees and clinicians. Results and recommendations will be shared with other network sites and publicized.

#### Vanderbilt University

##### Biobank description

The Vanderbilt biobank, BioVU, uses an "opt-out" model, based on the collection of DNA from discarded blood samples. Details of the system, including the methods of sample accrual, deidentification of patient records, and extensive internal and external review that the project continues to undergo, have been published elsewhere [22].

In brief, the approach adopted is based on federal Office for Human Research Protections (OHRP) guidelines (http://www.hhs.gov/ohrp/policy/cdebiol.pdf) stating that that use in research of discarded samples that are de-identified and not readily re-identified does not involve human subjects and so is not subject to the federal regulations for human subjects research (45 CFR 46, also called the Common Rule).

The Vanderbilt University Medical Center EMR has been developed as a clinical care and research tool over the past 15 years and covers all inpatient and outpatient data entry in the health system, including labs, drug ordering, and diagnostic imaging [23]. It includes >1.9 million records, and provides a platform for the development of tools, such as Natural Language Processing approaches, to optimally mine structured data and unstructured (free-form) text in the medical record. A key component of the BioVU project is the Synthetic Derivative (SD), a research-optimized copy of the Vanderbilt EMR from which identifiers have been removed.

BioVU acquires and deidentifies blood samples obtained in out-patient clinics and scheduled to be discarded by Clinical Pathology. The link between clinical information in the SD and the DNA sample is retained by labeling each sample and each entry in the EMR by a Research Unique Identifier (RUI) generated by the Secure Hash Algorithm (SHA-512), developed by the National Security Agency of the United States Federal Government (http://www.nsa.gov/ia/_files/os/redhat/rhel5-guide-i731.pdf). SHA-512 is a publicly available hash function, an algorithm that produces a string of 128 characters that is unique to a particular input; in other words, it will always generate the same output (RUI, in this case) given the same input (medical record number, in this case). Sample handling and de-identification procedures underwent extensive pre-launch evaluation that has been previously reported [22].

##### eMERGE project aims

*Phenotypes*

The primary site-specific phenotype that is being analyzed by GWAS in the Vanderbilt Genome Electronic Records (VGER) project, the Vanderbilt component of eMERGE, is duration of QRS complex on the surface electrocardiogram (ECG), a measure of cardiac conduction velocity. Natural language processing and other approaches are being used to identify ECGs designated as clinically normal in subjects without conditions thought to affect QRS duration, such as heart disease or certain drugs. The ECG is one of the most commonly-ordered tests and provides information on heart disease and on variation - even within the normal range - of key indices of cardiac function. Specifically, slow conduction enables abnormal heart rhythms, a major public health problem, so analysis of the duration of the QRS complex is a first step in identifying new pathways to arrhythmias.

The availability of genotypes across a large number of subjects with phenotype-rich records has enabled Vanderbilt investigators to start to explore the capabilities of a "Phenome-wide Association Study" (PheWAS) [23]. The BioVU ARRA supplement aims to extend this approach to other sites in eMERGE.

*Community Consultation*

The VGER aims related to community consultation are 1) to assess the ethical, scientific, and societal advantages and disadvantages of the BioVU model, and determine best practices for oversight, community involvement, and communication as the resource grows; and 2) to develop and evaluate formal privacy protection models for data derived from databanks and EMRs, establishing data sharing and integration practices.

Retrieved from http://www.mc.vanderbilt.edu/victr/dcc/projects/acc/index.php/About

## Current Status of the Network

### Choice of genotyping platform and QA

As of June 2010, all sites had received genotyping data from DNA samples that had been shipped to NHGRI-designated central genotyping laboratories at the Broad Institute and the Center for Inherited Diseases Research at Johns Hopkins University. The Illumina 660Wquad1a was selected as the genotyping platform for subjects of European ancestry and other racial/ethnic groups than African Americans, while the Illumina 1 M product is being used for African-American subjects. The distribution of samples across sites and primary phenotypes is included in Table 1. The process for quality control (QC) of the genotyping data is discussed at weekly teleconferences led by the Administrative Coordinating Center (ACC) with participants from NHGRI, NCBI, the genotyping centers, and the Genomics workgroup. The Genomics workgroup is developing a uniform QC pipeline for the eMERGE network that will be publicly available upon completion.

### Sharing of genotype and phenotype data

To compare phenotypes across sites, data sharing agreements have been initiated between the individual eMERGE sites and the consortium's ACC and between all of the individual sites. These allow sharing of de-identified ICD-9 codes to compare underlying population characteristics among the sites, and to share phenotypic and genotypic data to allow the combining of data to increase sample size and allow for replication. For example, additional dementia cases were provided by two sites to augment the cases at GHC.

One example of increased statistical power afforded by the network is presented in Table 2 for one of the secondary outcomes (diabetic retinopathy) that leverages genotyping already performed for one of the primary eMERGE phenotypes.

**Table 2:** Sample size estimates for GWAS of diabetic retinopathy with power estimates for the individual sites and with combined data

**eMERGE site**	**Estimated number of diabetic retinopathy cases and controls**	**Power for GWAS, p < 10**^**-8**^**, MAF = .10 OR = 2.5**
Marshfield	367/569	.424
Northwestern	150/1262	.243
Mayo	83/3412	.101
Group Health	324/667	.449
Vanderbilt	260/500	.270
Combined	1184/6410	.999

Another example of cross-site collaboration is in the QA/QC of all of the GWAS genotype data. The ACC performs quality assurance for all genotyping data. All sites submit phenotypic data to dbGAP at the same time they submit DNA samples for genotyping to allow adequate time for data cleaning prior to the genotyping results being available. The ACC has ensured common definitions and coding for phenotype variables being deposited in dbGAP. As mentioned above, the ACC also facilitates the QA/QC of these data in collaboration with the other components of the network. This ensures that accurate genotype and phenotype data will be deposited to dbGaP.

**Figure 2:**
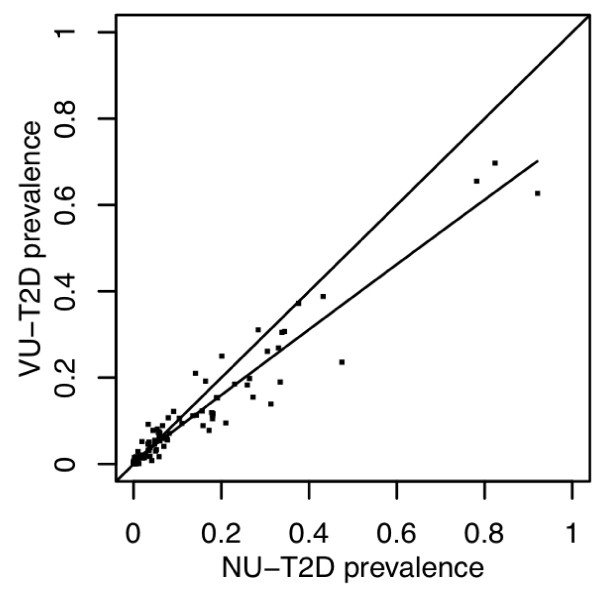
**Comparison of ICD9 Codes Between two sites for patients with Type 2 Diabetes**. The graphic shows the prevalence of ICD-9 codes, grouped into ICD-9 code sections (n=120). The linear regression line drawn between these two populations show that these two groups are similar, with a slope slightly favoring a higher prevalence of more codes at Northwestern (NU) than Vanderbilt (VU).

Another example of early cross-site integration is a study being performed by the ACC comparing prevalent ICD-9 codes across network sites for similar and different phenotypes to assess the comparability of large populations taken from different institutions (Figure 2). The goal of this research is to develop a metric to quantify the similarity and differences of two populations across all diseases (using the range of ICD-9 codes). In addition, this method should allow rapid elucidation of particular differences in the populations. These results are showing that different institutions employing similar phenotype definitions produce populations with similar comorbid disease distributions, while different phenotypes tend to be more dissimilar.

### Choice of cross-network phenotypes

A ranking exercise was developed for network-wide phenotypes and completed by all five sites. Elements of the ranking included the importance of the scientific question, whether GWAS had been performed for that outcome before, and the relative ease or difficulty of developing accurate electronic phenotyping algorithms appropriate to EMR-derived clinical data. Autoimmune hypothyroidism was the first cross-network phenotype selected to meet these criteria with sufficient numbers of cases and controls across the network. However, an examination of records associated with samples already targeted for GWAS identified several thousand subjects with hypothyroidism. This finding further reinforces the potential that generating dense genomic information across subjects with EMRs will represent an important and reusable resource for discovery and validation in genome science

### Goals for network collaboration

After network sites were selected, and initial progress was made on the site-specific goals, the External Scientific Panel (ESP) reviewed the program and generated a set of further goals for the network as a whole. As a result, work at each network site has been extended to include cross-site comparisons and sharing of new tools and approaches. Examples include, in addition to hypothyroidism mentioned above, other commonly acquired clinical laboratory values as continuous traits.

For phenotypes already subjected to numerous GWAS, such as myocardial infarction and type 2 diabetes, investigators were encouraged to identify unique or compelling features that could be added based on the ability to mine the entire EMR, and/or to focus on minority subjects, if available. Vanderbilt and Northwestern partnered to include a large sample of African Americans in a study of type 2 diabetes.

Each site has been encouraged to address issues of non-genetic, environmental exposures and their impact on specific phenotypes (e.g. baseline socio-economic status, smoking) and determine to what degree these measures can be extrapolated from the EMR. Validation of the accuracy of smoking data from EMRs is being undertaken at Vanderbilt, Mayo Clinic and Marshfield and Marshfield has added smoking history to the vitals section of their internally-developed EMR.

The ESP indicated that feedback to the EMR community is essential, both to private companies that produce widely-used EMR software and others working on EMRs. This is especially true for important phenotypes that can be broadly defined, as that will make it easier to extend the methods developed to many more sites. Marshfield will be working to implement eMERGE recommendations in their internally developed EMR.

Vanderbilt is adding a pediatric component to their biobank and will examine and report on consent and proxy issues and share lessons learned with the network.

Phenotyping efforts will be expanded to include drug responses and their relationship to genome variation. Drug responses are also likely to be important in defining other eMERGE-related disease phenotypes (such as steroid use and its relationship to conditions such as cataract sub-type and asthma response).

All sites have developed a list of best practices to be shared with the network and the wider research community (Table 3).

**Table 3:** Best practices being developed by the eMERGE sites

**eMERGE site**	**Best practices**
Marshfield	Enhance internal EMRs to capture data in a structured format. This may involve changing existing input points in the record. Information validated against questionnaire data where applicable; Develop and evaluate a computer-based consenting process along with revisions to the current written informed consent document for our general biobank; Development of validated electronic algorithms for cataract, HDL, and diabetic retinopathy
Mayo Clinic	Manual abstraction vs. EMR-based algorithms: virtually all algorithms ultimately are dependent upon unstructured data; develop criteria for standardizing data dictionaries and best practices for handling missing data elements; community engagement survey instrument & educational video to educate community regarding biobank and community engagement processes; develop institutional policy and procedure for sharing of GWAS data; assess phenotyping heterogeneity from the EMR
Northwestern	Informatics: Identify shortcomings of data capture from routine clinical care and repurposed for research; Develop and implement common standards for formatting and sharing data; Community engagement: Develop model consent language; Summarize community engagement efforts around data sharing in our population; Genomics: Develop process for GWAS data certification review and approval; Other/general: Develop best practices for interacting with IRBs around biorepository formation and ongoing consultation
Group Health	Mapping the electronic derived cases vs. 'research quality' (e.g. dementia). How to handle cases from different sources; Use of "low tech" methods to extract NLP information; identify participant-centered best practices regarding consent from existing cohorts; develop recommendations for institutions, investigators re consent, data sharing, other issues with GWAS and related research (products from a consensus panel process)
Vanderbilt	Identify shortcomings and enhance internal EMRs to capture data in a structured format; develop methods for assessing/labeling certainty of data shared to public databases; create a description of the various analogs to human subjects biobanking in a non-human subjects model
Administrative Coordinating Center	Creation of a library of searchable phenotype algorithms plus associated metadata; creation of educational materials on genomic data privacy for IRBs and other regulatory decision makers; develop a re-identification risk framework for biomedical data to be shared to dbGaP

## Summary

### Lessons learned

Many challenges have been identified and some have been overcome, providing a set of information that can be used by groups wanting to undertake similar research. The biggest lessons learned to date have come from the informatics arena. With the major task to develop validated algorithms to define phenotype from EMRs, investigators quickly confirmed that diagnostic codes are insufficient on their own for research-quality case and control classification. Each EMR is unique and EMR systems do not necessarily contain all data elements necessary for a given case definition. Differences in site populations suggest that the cohorts selected may not be clinically comparable for some conditions being studied. This is currently being evaluated by measuring the distribution of disease codes that co-occur with the primary disease phenotype that is the focus of a particular GWAS (for example, the frequency of heart disease, stroke and renal failure disease codes in cohorts selected because the individuals have diabetes). Furthermore, if the health system with the biobank is not the primary health care provider, information necessary to assign case and control status may be lacking because of care received elsewhere that is not documented in the EMR. Analysis of free text can be very difficult for electronic data extraction, but has been shown to be essential for case/control classification for several of the phenotypes. A useful strategy is to engage content experts early in algorithm development. A common validation methodology to determine the predictive value of the computerized cohort selection algorithms needs to be employed. Not surprisingly, achieving the required consensus on data standards and data dictionaries is time consuming but essential to the analysis of pooled data from heterogeneous systems. Data sharing is facilitated where common or similar institutional policies and procedures are developed.

Ethical issues that have been identified include whether the original consent allowed for sharing of data with dbGaP or investigators from other institutions, and whether the original consent allowed for the study of more than one phenotype. Investigators at Group Health and the University of Washington undertook a sub-study of reconsent of their subjects to deposit deidentified research data in dbGaP [24,25].

Potential and real benefits of the network have been identified. The power of the network includes collaborations with other experts in the field, sharing of protocols, data/drug mining lessons learned that can be implemented in other EMRs, and the development of well validated electronic phenotyping algorithms that can be used for case/control classification and for identification of potential subjects for study/trial recruitment.

### Next steps in Current Funding Cycle

In addition to the outcomes already mentioned, the eMERGE network is developing and disseminating best practices recommendations in the areas of electronic phenotyping and community consultation related to biobanking and data sharing. The wealth of phenotypic information in the EMRs is being mined for additional GWAS outcomes in the genotyped subjects.

### Future Directions for the Network

Plans are underway to expand the network in diversity of populations and incorporation of GWAS findings into clinical care.

### Summary

The eMERGE network is advancing our knowledge of best practices related to the linking of biobanks with data from electronic medical records to facilitate genomic discoveries in an efficient manner. As biobanks are developed within health care settings, the lessons learned from the eMERGE network can be applied to these settings to further scientific discoveries in a timely fashion.

## List of Abbreviations

ABI: ankle brachial index; ACC: Administrative Coordinating Center; ACT: Adult Changes in Thought study; AD: Alzheimer's Disease; ARRA: American Reinvestment and Recovery Act; CASI: Cognitive Abilities Screening Instrument; CTSA: Clinical and Translational Science Award; dbGaP: database of genotypes and phenotypes; DNA: deoxyribonucleaic acid; DSM: Diagnostic and Statistical Manual; ECG: electrocardiogram; EDW: enterprise data warehouse; ELSI: ethical, legal and social issues; eMERGE: electronic Medical Records and Genomics; EMR: electronic medical record; ESP: External Scientific Panel; GH: Group Health, GWAS: genome wide association study; HAAS: Honolulu Asia Aging Study; HDL: high-density lipoprotein; ICD: International Classification of Diseases; ICR: intelligent character recognition; MAF: minor allele frequency; NHGRI: National Human Genome Research Institute; NIH: National Institutes of Health; NINCDS-ADRDA: National Institute of Neurological and Communicative Disorders and Stroke - Alzheimer's Disease and related Disorders Association; NLP: natural language processing; NUMC: Northwestern University Medical Center; OR: odds ratio; PAD: peripheral arterial disease; PheWAS: phenome-wide association study; PMRP: Personalized Medicine Research Project; QA: quality control; QC: quality control; RBC: red blood cells; RFA: Request for Applications; RUI: research unique identifier; SD: synthetic derivative; SHA: secure hash algorithm; T2D: type 2 diabetes; TSH: thyroid stimulating hormone; VGER: Vanderbilt Genome Electronic Records project; VUMC: Vanderbilt University Medical Center; WBC: white blood cells

## Competing interests

The authors declare that they have no competing interests.

## Authors' contributions

Catherine A. McCarty is the site PI at Marshfield and took the lead to draft the manuscript, Rex L. Chisholm is the site PI at Northwestern, Christopher G. Chute is the site PI at Marshfield, Iftikhar Kullo is leading the phenotyping effort for the primary phenotype at Mayo, Gail Jarvik is the Co-PI for the group Health site, Eric B. Larson is the site PI at Group Health, Rongling Li is the NHGRI Project Officer, Daniel R. Masys is the PI of the Administrative Coordinating Center

Marylyn D. Ritchie is responsible for the QA of the genotype data for all sites, Dan M. Roden is the site PI at Vanderbilt, Jeffery Struewing was the initial NHGRI Project Officer, and Wendy A. Wolf is the Program manager at Northwestern, providing input into study design. All authors provided input to the study design at their respective sites and the overall network objectives, and all authors read and approved the final manuscript.

## Pre-publication history

The pre-publication history for this paper can be accessed here:

http://www.biomedcentral.com/1755-8794/4/13/prepub
